# A Comparison between heat transfer performance of rectangular and semicircular tubes considering boundary effects on Brownian motions in the presence of Ag / water nanofluids: Applicable in the design of cooling system of photovoltaic cells

**DOI:** 10.1371/journal.pone.0180883

**Published:** 2017-07-28

**Authors:** Amin Jafarimoghaddam, Sadegh Aberoumand

**Affiliations:** 1 Aerospace Engineering Department, K. N. Toosi University of Technology, Tehran, I.R. Iran; 2 Independent Researcher, Tehran, Iran; North China Electric Power University, CHINA

## Abstract

The present study aims to experimentally investigate heat transfer performance of rectangular and semicircular tubes in the presence of Ag / water nanofluids. The nanoparticles of Ag (silver) were used in seven different volume concentrations of 0.03%, 0.07%, 0.1%, 0.2%, 0.4%, 1% and 2%. The experiment was conducted in relatively low Reynolds numbers of 301 to 740. A heater with the power of 200 W was used to keep the outer surface of the tubes under a constant heat flux condition. In addition, the rectangular tube has been designed within the same length as the semicircular one and also within the same hydraulic diameter. Moreover, the average nanoparticles size was 20 nm. The outcome results of the present empirical work indicate that, for all the examined Reynolds numbers, the semicircular tube has higher convective heat transfer coefficient for all the utilized volume concentrations of Ag nanoparticles. The possible reasons behind this advantage are discussed through the present work mainly by taking the boundary effect on Brownian motions into account. Coming to this point that the conventional design for cooling system of photovoltaic cells is a heat sink with the rectangular graves, it is discussed that using a semicircular design may have the advantage over the rectangular one in convective heat transfer coefficient enhancement and hence a better cooling performance for these solar cells.

## Introduction

These two last decades have been an exhibition of the applications of nanofluids. Since, the former routes for increasing the heat transfer (ex. Increasing the heat transfer surface and so forth) have proved themselves financially inefficient, nanoparticles were employed and examined in the base fluids in order to substantially enhance the heat transfer in a way with the most comfort in financial and manufacturing considerations. Many of investigations on nanofluids have been aimed to calculate the convective heat transfer in variety of applications (see [1–5]). The performance of different combinations of nanoparticles and base fluids have been tested and accordingly, correlations have been proposed for predicting Nussulet number [3–8]. One of the recent applications of nanofluids is to use them in the cooling system of photovoltaic cells. Since, the goal of the present empirical work is mainly to investigate an alternative design for the cooling system of the recent solar cells; it is worth to first provide a brief review on the recently used mechanisms for cooling the photovoltaic cells. One of the challenges to reaching an optimized design for the photovoltaic cells is to decrease the temperature of their working surface (the surface in which the ions move through the electrolyzed media). So, many studies have been done so far in order to increase the heat transfer rate from the PV cells [9–15]. Among the proposed mechanisms, heat sinks are most widely studied, especially because of their relatively low thermal resistance [15]. Therefore, there are lots of applications of micro and milli heat sinks on the removal of considerable heat from a small point or area. This applicability in the removal of heat has attracted many studies on milli and micro heat sinks (it is worth to note that channels with the hydraulic diameters less than 1 milli meter are considered as micro channels). Energy and Exergy analysis of a micro channel (in the constant mass rate of air) for cooling the PV cells have been done by Agrawal et al. [9]. An optimization on the performance of the cooling system of PV cells has been proposed by Karathanassis et al. [10] by introducing two micro channel configurations. In that work, planar fins were attached to the micro channels in order to increase the efficiency of the cooling system. Moreover, Ramos-Alvarado et al. [11] has reported the effectiveness of using liquid as the working fluid in micro channels. Demanding more efficiency for the cooling systems of PV cells pushed many of researchers to apply nanofluids. So, many attempts have been conducted so far in order to examine the ability of nanofluids (mainly in low volume concentrations) to enhance the efficiency of heat transfer rate in PV cells [14, 15]. Saidur et al. [13] examined the applicability of Aluminum / water nanofluid for cooling the PV panels. Tyagi et al. [14] conducted an experimental investigation on the impact of different diameter of nanoparticles on the performance of nanofluids in increasing the heat transfer rate. They reported that different nanoparticle sizes will not considerably affect the heat transfer. Taylor et al. [16] compared the performance of nanofluids with the conventional methods in increasing the heat transfer rate and proved that the nanofluids are more effective than the conventional routes. Karami et al. [15] conducted an experiment with the Boehmite / water nanofluid in a Perspex plane with 40 parallel micro channels. In that experiment, the micro channels have been configured with hydraulic diameter of 783 μm, 24 cm length, 1.8 mm width and 500μm height. These Perspex planes were attached to the back surface of the PV panels. They compared water (as the base fluid) with three weight concentrations of 0.01%, 0.1% and 0.3%. They reported that the average temperature of the PV panel surface was decreased from 62.29°C to 32.5°C for different inlet volume rates up to 300 ml/ min and Boehmite / water nanofluid with the weight concentration of 0.01%. Finally, they reported that for this weight concentration and the inlet volume rate of 300 ml/ min (as the best case of that study), the electrical efficiency of the PV cells increased by 27%. Having this review, the present experimental work has been done mainly to introduce a promising scheme for designing milli channels which has a better effect on heat transfer rate and subsequently may be considered as a replacement for the conventional scheme of heat sinks (heat sinks with rectangular cross sections). Ag/ Water nanofluids were selected as the multi- phase media to compare the efficiency of two different geometries of milli channels in convective heat transfer coefficient in the presence of nanofluids. The first geometry was the well- known conventional one (single milli channel with a rectangular cross section) and the second one was a milli channel with a semicircular cross section. The first shape indicates single unit of a milli channel heat sink design and the second shape simply represents a unit of a milli channel heat sink in which the graves are annularly manufactured. Moreover, the experiment conditions for the two applied geometries (which will be specified in the next sections) allowed us to seek for the possible reasons behind the outcome results through the difference between the patterns of Brownian motions in the two geometries.

## Materials and methods

### Nanofluids preparation

**Fig 1:**
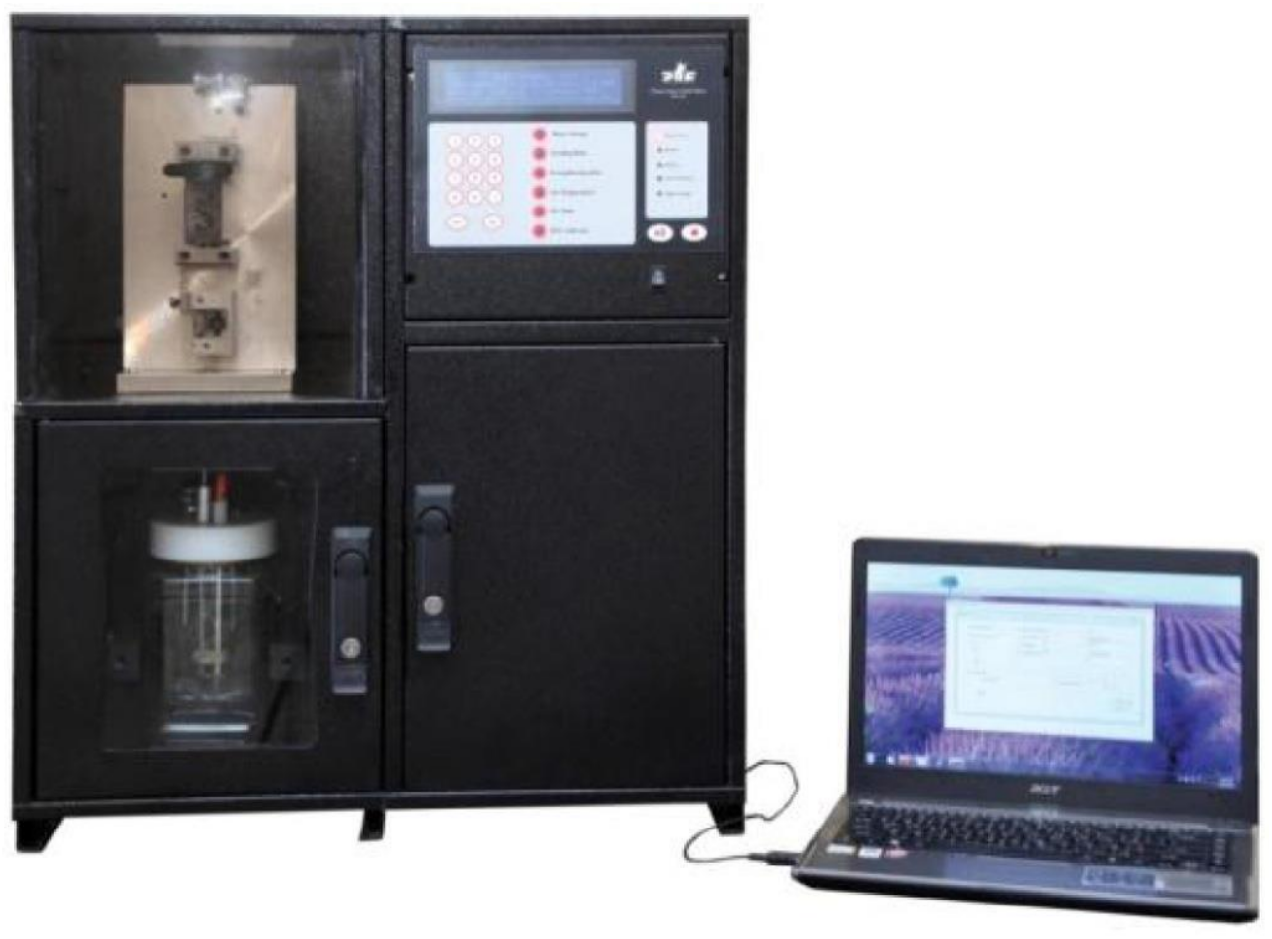
PNC1K device.

Electrical Explosion Wire (*E*.*E*.*W*) as a one- step method was used for the preparation of nanofluids. In this method by applying extra high electric voltage and current, the primary bulk wire converts into the nanoparticles via pulse explosive process. It is necessary to explain that, another special feature of this system is the possibility of adding a surfactant to the liquid. So, the nanofluid produced through this method, can remain stable for a long time. Among all of the existing methods in the production of metal nanoparticles, electrical explosion method is the most economical and industrial one. Furthermore, this method has been taken into account in the previous works by authors using PNC1K device [1–4]. An image of PNC1K device can be seen in Fig 1.

### Experimental apparatus

Since the main scope of this study is evaluating the heat transfer performances while using different tube geometries, an experimental procedure was set up to simulate a photovoltaic system. For this, a heat flux of 200 W was applied which can be considered as the heat flux exists from PVT cells.

**Fig 2:**
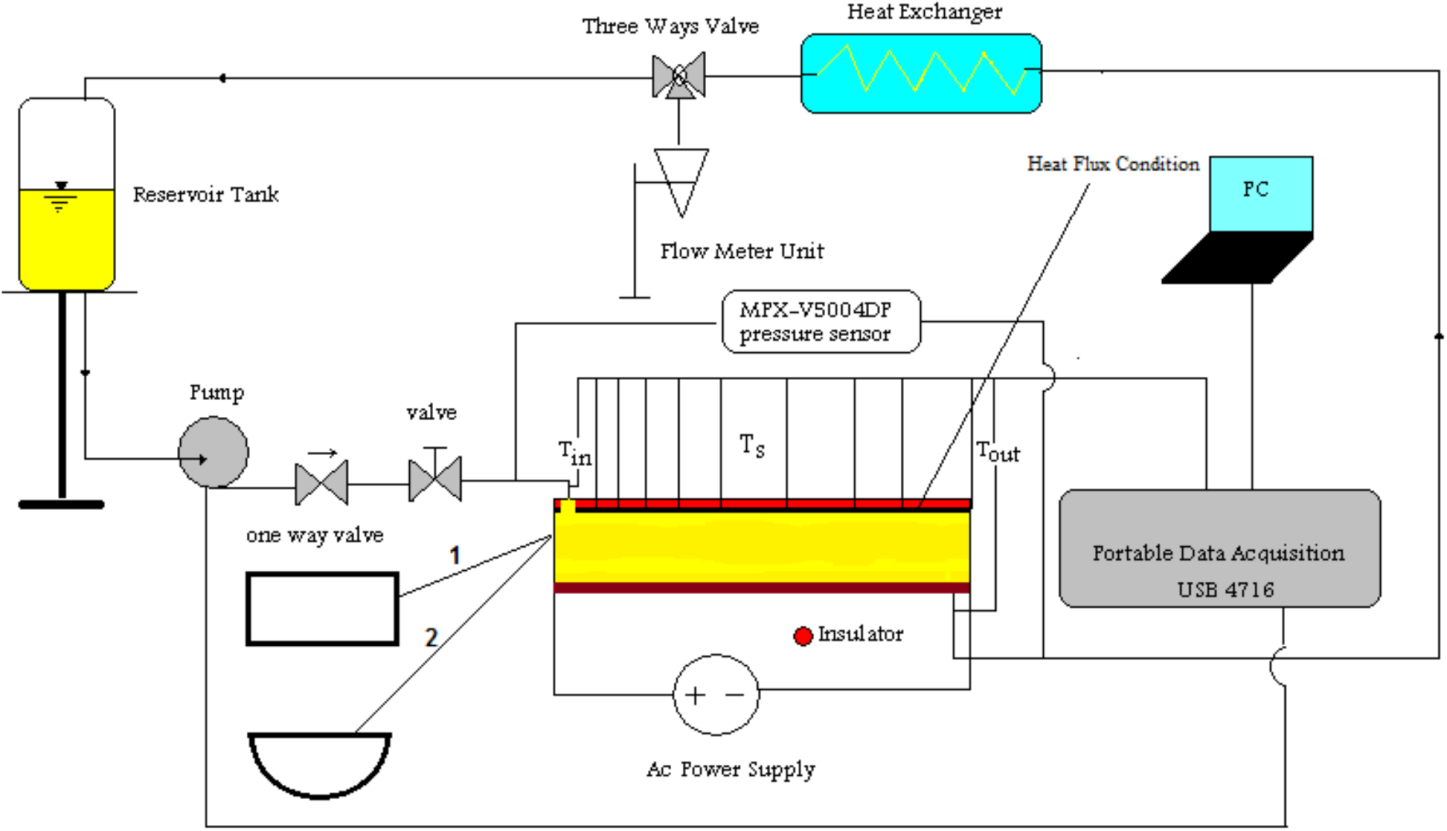
Schematic of the experimental setup.

The experimental setup consists of a flow loop, including a pump, a nanofluid reservoir, a gate valve, a non return valve, test section, RTD PT100 temperature sensors, MPX-V5004DP pressure sensor, data acquisition USB 4716 and a three- way valve. An electrical wire coil was attached to the upper surface of the tubes, which was linked to AC power supply. This power supply satisfies the constant heat flux rate of 200 W. Two calibrated temperature RTD PT100 sensors are installed in entrance and exit the test section for measuring inlet and outlet temperature of fluids. As previously discussed, two test cases were experimented with. The first was a simple duct (rectangular tube) with 0.5m length and 9.4 mm for the hydraulic diameter while the thickness of the duct was about 0.3 mm. The second test case was a tube with a semicircular cross section. The length, thickness and the hydraulic diameter of the second test case were reasonably corresponded to the first one. Test cases were copper plates manufactured by bystronic BYSTAR 3015 model of CNC. The two applied test cases are indicated by the numbers of 1 and 2 within the schematic provided in Fig 2. Moreover, for each of the test cases, the plane which was connected to the heater was finely isolated in order to be certain that the heat dissipation is ignorable during the experiment.

Eleven RTD PT100 sensors were employed to measure the wall temperature of the test section and all of them welded optionally at 1, 5, 10, 15, 20, 25, 30, 35, 40, 45 and 49 cm of axial distance. A 1 litter vessel and stop watch accurate to 0.001 s was used to measure the flow rate. A heat exchanger unit was settled in after the test section. Fluid turns back to the pump from the fluid reservoir, then it pumps to the test section and bulk temperatures and wall temperatures are measured with sensors. In addition, all the measurements were done when the flow reached its steady state mode and moreover, for being sure about the absorption of the nanoparticles through the tubes due to the low Reynolds numbers, by taking samples from the reservoir tank and measuring their specific weights, the setup was checked for each of the examined test case.

## Data acquisition

### Nanofluids stability

Before employing nanofluids for evaluating heat transfer enhancement, it is essential to be sure about the stability of the suspended nanoparticles inside the base fluid. Stability check is possible by considering zeta potential as an index for assessing the stability. This index simply relates the stability of nanofluids to the electro- static repulsions of the particles surface. So, having the index for a specific nanofluid greater than 30mV, the nanofluid is mainly categorized among the stable types. While the values of this index smaller than 20 mV represents a weak stability of the nanofluids. Zetasizer Nano ZS made by Malvern, Britain utilizes a combination of electrophoresis and laser Doppler velocimetry for measuring the nanoparticles velocities while applying electrical field. In the next stage, having the nanoparticles velocity and the dielectric strength of the working fluid, Henry equation is applied and the Zeta potential will be evaluated by applying Smoluchowski equation. All the mentioned equations and related explanations are presented in the study of P. Leroy et al [17]. The impact of surface conductivity of TiO_2_ nanoparticles on Zeta potential is presented in their study. Taking the Ag nanoparticle shape which is spherical and is similar to TiO_2_ into account, their discussion can be used as the explanations behind of measuring Zeta potential in this work.

**Fig 3:**
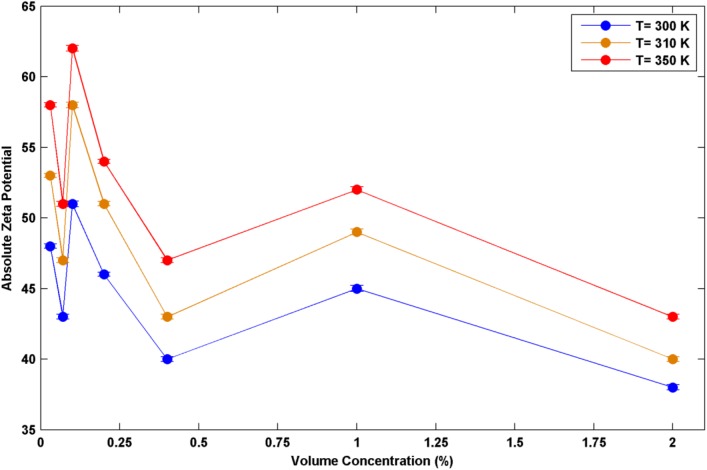
Absolute zeta potential in different volume concentrations.

In the present work, Zeta potential is measured for three optional temperatures of 300, 310 and 350 K and all the applied nanoparticles concentrations using Zetasizer Nano ZS made by Malvern, Britain and the results are illustrated in Fig 3.

### Uncertainty

Any experiment has uncertainties mainly due to the inherent errors of the instruments used in the experiment. Similar to the previous works by authors [1–5], Coleman and Steele uncertainty method was employed in the present work to evaluate uncertainties for convective heat transfer coefficient and friction factor. Based on this method, the errors were calculated to be 2.8% for convective heat transfer coefficient and 3.7% for friction factor which can be considered as the reliable errors.

### Thermo- physical properties of nanofluids

**Fig 4:**
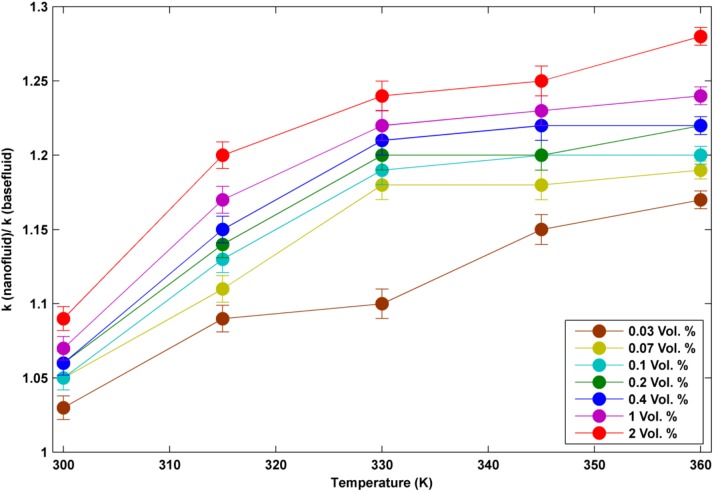
k_nf_/ k_bf_ in different temperatures.

**Fig 5:**
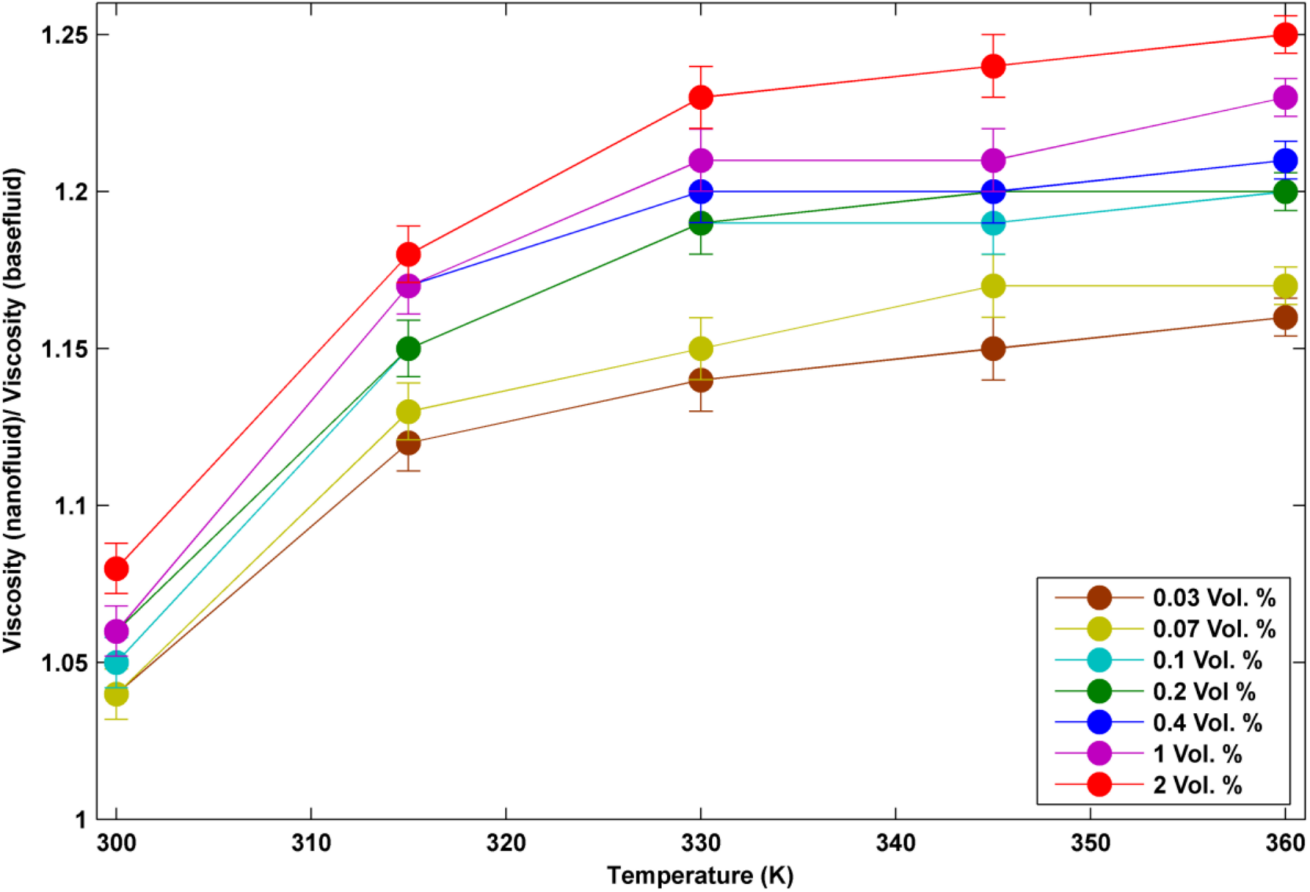
μ_nf_/μ_bf_ in different temperatures.

**Fig 6:**
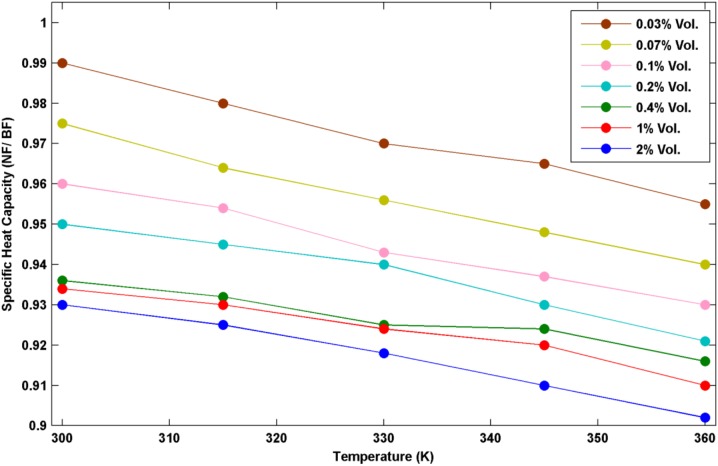
Cp_nf_/ Cp_bf_ in different temperatures.

In order to quantify the specific heat capacity (c_p_) in different temperatures of nanofluids and pure water, a differential scanning calorimeter (DSC F3 Maia, manufactured by NETZSCH-Germany) was used. SVM3000 devise was used to determine the density in different temperatures and volume concentrations of nanofluid. The thermal conductivity and viscosity of nanofluids and pure water were measured by KD2 thermal properties analyzer and Brookfield viscometer (DV-II + Pro Programmable Viscometer), respectively. Thermo- physical properties of the utilized nanofluids in different volume concentrations and temperatures are shown in Figs 4–6.

As it can be seen from these plots, the two factors of k_nf_/ k_bf_ and μ_nf_/ μ_bf_ enhance with the increase of temperature while C_pnf_/ C_pbf_ decreases with the increase of temperature. Thermal conductivity enhancement ranges from around 5% to 25% in different conditions of temperatures and volume concentrations. As all data have been acquired from the accurate experiments, some data seems to be unmatched to the total pattern. This difference is likely because of the low volume concentration which affects the thermal conductivity weakly.

## Data analysis

### Factors determination

By considering the energy balance on a differential control volume of the fluid inside the tube:

$$\frac{dT_{m}}{dx}=\frac{q^{"}P}{\dot{m}c_{p}}=\frac{P}{\dot{m}c_{p}}h(T_{s}(x)-T_{m}(x))\Rightarrow T_{\text{m}}(\text{x})={\text{T}}_{\text{mi}}(\text{x})+\frac{q^{"}Px}{\dot{m}c_{p}}$$ (1)

Where $\dot{m}$ is the mass flow rate and P is the upper edge of the tubes which was entirely attached to the heater. Specifically, the cross sections of two tubes had an equal hydraulic diameter and for the rectangular tube, the length of the rectangle was about 0.8 of the semicircle diameter. As it is clear, the size of the upper edge of the tubes is different, but by isolating the semicircular tube from the upper side, the factor P in Eq 1 was precisely kept the same for the two tubes. So, by this way, one can simply study the effects of external dynamic patterns (Brownian motions) on convective heat transfer coefficient enhancement. On the other hand, we were sure that increasing the heat transfer surface will not affect the outcome results of the present experiment. In addition, in Eq 1, T_s_, T_m_ and c_p_ are the surface temperature, mean temperature and specific heat capacity respectively. x is distance from the tube inlet. To calculate the convective heat transfer coefficient:

$$h(x)=q^{''}/\left(T_{s}(x)-T_{m}(x)\right)$$ (2)

Reynolds and friction factor are defined as follow:

Re=4m˙/πDhμf=Δp/((1/D)ρ(v2/2)) (3)

## Results and discussion

Based on the acquired data, in the two studied geometries, convective heat transfer coefficient (h) increased by addition of nanoparticles to the base fluid (see Table 1; in which subscribes of 1 and 2 indicate the rectangular tube and the semicircular tube, respectively). This increase occurred for all the applied nanoparticle concentrations and for the two applied geometries. So, it is worth to first provide a discussion on the possible reasons of the enhancement in convective heat transfer coefficient due to the addition of nanoparticles. The possible reasons behind the advantage of semicircular tube over the rectangular one by taking the concept of Brownian motions into account will be discussed.

**Table 1:** The most important data from the present experiment.

No.	Volume Concentration%	h_2_/ h_1_ (10W/ m^2. K)	Re	No.	Volume Concentration%	h_2_/ h_1_ (10W/ m^2. K)	Re
**1**	0	24.3/24.3	301	37	0.2	31.4/27.4	301
**2**	0	25.8/25.7	350	38	0.2	35.6/28.7	350
**3**	0	26.4/26.3	403	39	0.2	37/29.4	403
**4**	0	27.9/27.9	457	40	0.2	38.2/29.6	457
**5**	0	29.4/29.4	520	41	0.2	39.1/33.2	520
**6**	0	32.1/31.2	583	42	0.2	43.9/37.4	583
**7**	0	36.2/36.1	633	43	0.2	47.5/40.2	633
**8**	0	38.4/38.4	690	44	0.2	49.9/44.3	690
**9**	0	41.3/41.3	740	45	0.2	50.1/45.9	740
**10**	0.03	26.3/25.9	301	46	0.4	32/27.7	301
**11**	0.03	27.4/26.1	350	47	0.4	36.1/29	350
**12**	0.03	29.3/26.8	403	48	0.4	37.3/32.3	403
**13**	0.03	30.1/27.9	457	49	0.4	38.4/33.1	457
**14**	0.03	32.2/29.8	520	50	0.4	39.5/33.9	520
**15**	0.03	34.6/31.7	583	51	0.4	44.1/38.4	583
**16**	0.03	38.7/36.1	633	52	0.4	47.9/38.9	633
**17**	0.03	42.3/38.5	690	53	0.4	50.2/42.3	690
**18**	0.03	43.4/41.5	740	54	0.4	50.6/47.4	740
**19**	0.07	29.4/26.2	301	55	1	33.1/28.9	301
**20**	0.07	32.1/27.1	350	56	1	36.5/33.4	350
**21**	0.07	34.8/27.8	403	57	1	37.8/35.2	403
**22**	0.07	36.1/28.3	457	58	1	39.1/37.4	457
**23**	0.07	38.1/30.4	520	59	1	40.2/38.5	520
**24**	0.07	40.2/33.1	583	60	1	44.4/42.9	583
**25**	0.07	43.5/36.8	633	61	1	48.2/46.9	633
**26**	0.07	46.2/40.1	690	62	1	50.3/48.7	690
**27**	0.07	48.3/43.7	740	63	1	50.7/49.9	740
**28**	0.1	30.5/26.8	301	64	2	33.1/32.9	301
**29**	0.1	33.4/27.8	350	65	2	36.5/36.4	350
**30**	0.1	35.6/28.4	403	66	2	37.9/37.7	403
**31**	0.1	37.1/29	457	67	2	39.2/39	457
**32**	0.1	38.9/32.1	520	68	2	40.1/39.9	520
**33**	0.1	42.5/36.5	583	69	2	44.4/44.1	583
**34**	0.1	45.6/39.1	633	70	2	48.3/48.2	633
**35**	0.1	48.3/43.1	690	71	2	50.3/50.2	690
**36**	0.1	49.6/43.9	740	72	2	50.6/50.3	740

As previously noted, two aims were targeted during the present work. One was to compare the performance of the two mentioned geometries (rectangular tube and semicircular tube) in convective heat transfer coefficient in the way which could be applicable in the design of cooling system of photovoltaic cells. Secondly, by applying the same hydraulic diameters for the tubes and applying the same factor P for the two tubes, it was promising that we could also proceed in studying the effectiveness of Brownian motions on heat transfer coefficient enhancement.

Reynolds number is an invariant for Navier Stokes equations which particularly identifies the different flow regimes occurring by the fluid flow. But in the presence of nanoparticles, the dynamics of flow will be affected and then the imposed slip velocities by nanoparticles may affect the constraints of the momentum equations. Then, since the hydraulic diameter is developed as a guarantee for the dynamic equivalence in a fully developed pipe flow, may not be dominant in the presence of nanoparticles. Therefore, we have deliberately kept the cross sections of the tubes equivalent as well as the hydraulic diameter. So there would be more assurance that we have precisely conducted the two experiments dynamically equivalent.

Having a greater inlet flow rate (and may higher inlet Reynolds number), convective heat transfer coefficient will increase due to the shrinking of the thermal boundary layer. But in a certain Reynolds number, it is more convenient to study the effect of volume concentration on convective heat transfer coefficient enhancement by the macroscopic theory for the forced convective heat transfer. Based on this theory the convective heat transfer coefficient, may be formulated approximately as h = k_f_/d_t_, in which k_f_ is thermal conductivity and d_t_ is thermal boundary layer thickness. Both an increase in k_f_ or/and a decrease in d_t_ improve the convective heat transfer coefficient. An addition of nanoparticles to base fluid increases the thermal conductivity of nanofluid and this enhancement increases with the increase of particle concentration. The increase of the thermal conductivity increases the convective heat transfer coefficient subsequently. Coming to the behavior of the thermal boundary layer thickness, based on the simplified energy equation for incompressible and one- phase fluids, d_t_ might be reliant on the behavior of diffusivity factor which is mainly based on specific heat capacity and thermal conductivity.

Therefore, manipulation of these thermo- physical parameters may affect the diffusivity factor and subsequently convective heat transfer coefficient (h), which the diffusivity factor increases, so possibly decreasing the thermal boundary layer and vice versa. We insist “possibly”, because as the one- phase energy equation for incompressible flows is mainly a transport-like equation, the transport factors (velocity components) also play role. So, in a certain Reynolds number, by increasing the volume concentration of the suspended nanoparticles, viscosity may also increase and then for a higher volume concentration, we may have a higher inlet velocity due to the Reynolds equivalence. The higher velocity may be interoperated as a faster transport phenomena through the energy equation, hence a decrease in the thermal boundary layer thickness. So as is clear, behavior of h mostly relies on the combination of the mentioned parameters. But according to the outcome results of the present work, the manipulation of the thermal conductivity and the thermal boundary layer thickness have effectively improved the convective heat transfer coefficient for the two applied geometries (the two tubes with different cross sections).

**Fig 7:**
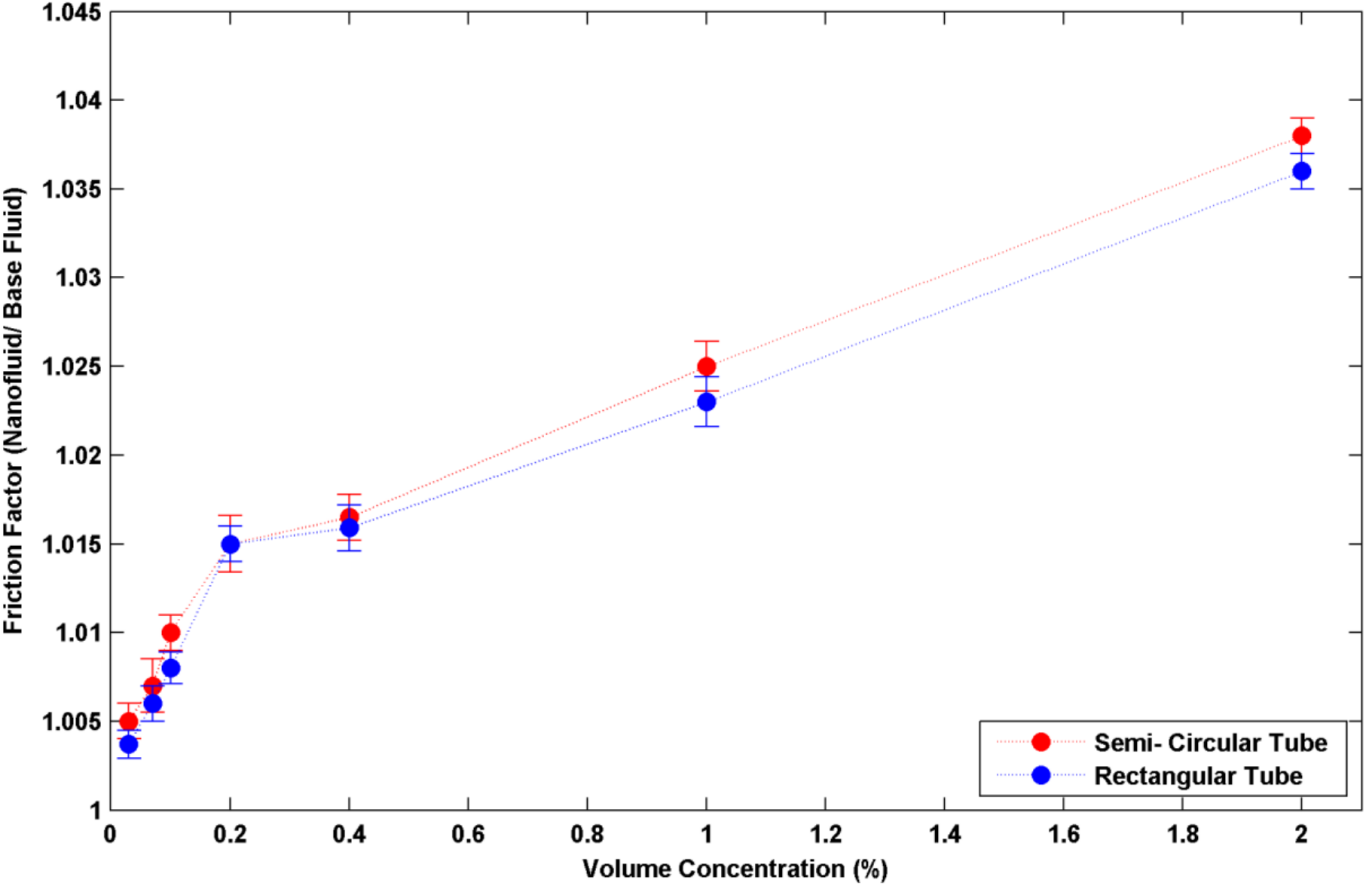
f_nf_/ f_bf_ in different volume concentrations of nanoparticles for the two applied geometry.

It is worth to note that a large number of researchers believe that particle size has a marginal effect (compared to Reynolds number and volume concentration) on convective heat transfer coefficient. This concept comes from the reality that nanofluids containing larger nanoparticles have a lower thermal conductivity; this effect should have led to a lower convective heat transfer coefficient. Wen and Ding [18] related this effect to the particle migration mechanism. According to this idea, large nanoparticles tend to migrate to the central part of the pipe, which could result in a particle depletion region with low viscosity at the near wall, hence a decrease in the boundary layer thickness. Also, small nanoparticles tend to be uniformly distributed over the pipe cross-section due to the Brownian motion. Hence, for a given average particle concentration, the wall region could have a higher solids concentration and hence a higher viscosity when the flowing nanofluids contain smaller nanoparticles. The combination of the above two opposite cited effects could also have been consequence for the observed marginal effect of nanoparticle size under the conditions of this investigation. It must be cited that, the proposed particle migration mechanism is a hypothesis and it may need further experimental works in order to more verification and confirmation. The effective viscosity increases by adding the nanoparticles to the base fluid. So, it is easily concluded that the fluid resistance for flowing, increases by having higher nanoparticle concentrations. This increase could be observed in the increase of friction factor. According to the acquired data of the present experiment, friction factor rises slightly for all the studied nanoparticle concentrations. As it is well known, this increase is counted as a weakness for nanofluid applications. But based on the outcome results of this study, this increase is almost negligible for all the cases. In which, for the worst case, f_nf_/ f_bf_ did not exceed than 1.04. This means that for all the studied cases, there will not be the need for considerably extra pump power supply to guarantee the nanofluids circulation. The results for the friction factor (f_nf_/ f_bf_ was averaged over the applied Reynolds numbers for each particular volume concentration) are shown in Fig 7.

**Fig 8:**
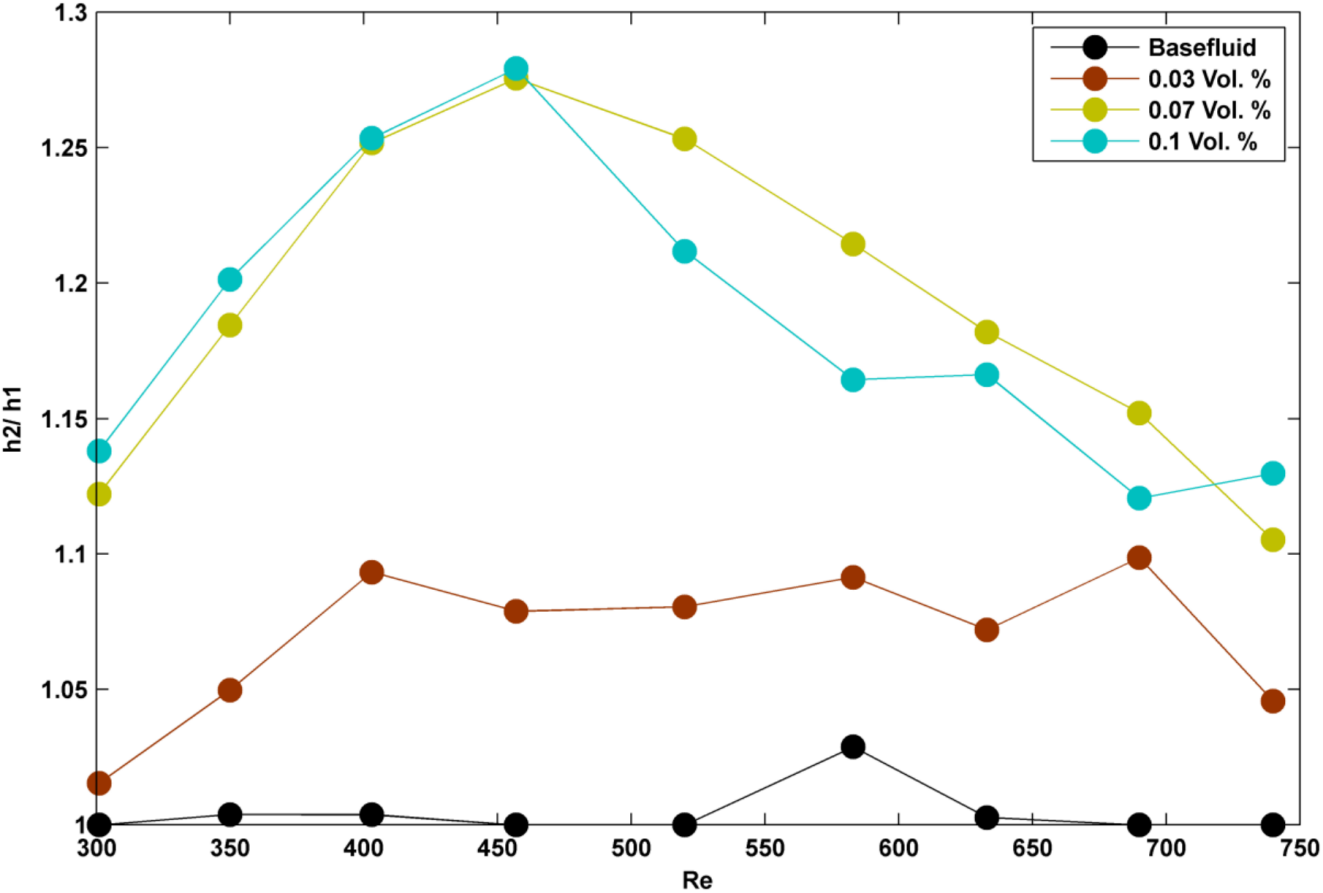
h_2_/ h_1_ in different Reynolds numbers and for lower volume concentrations of nanoparticles.

**Fig 9:**
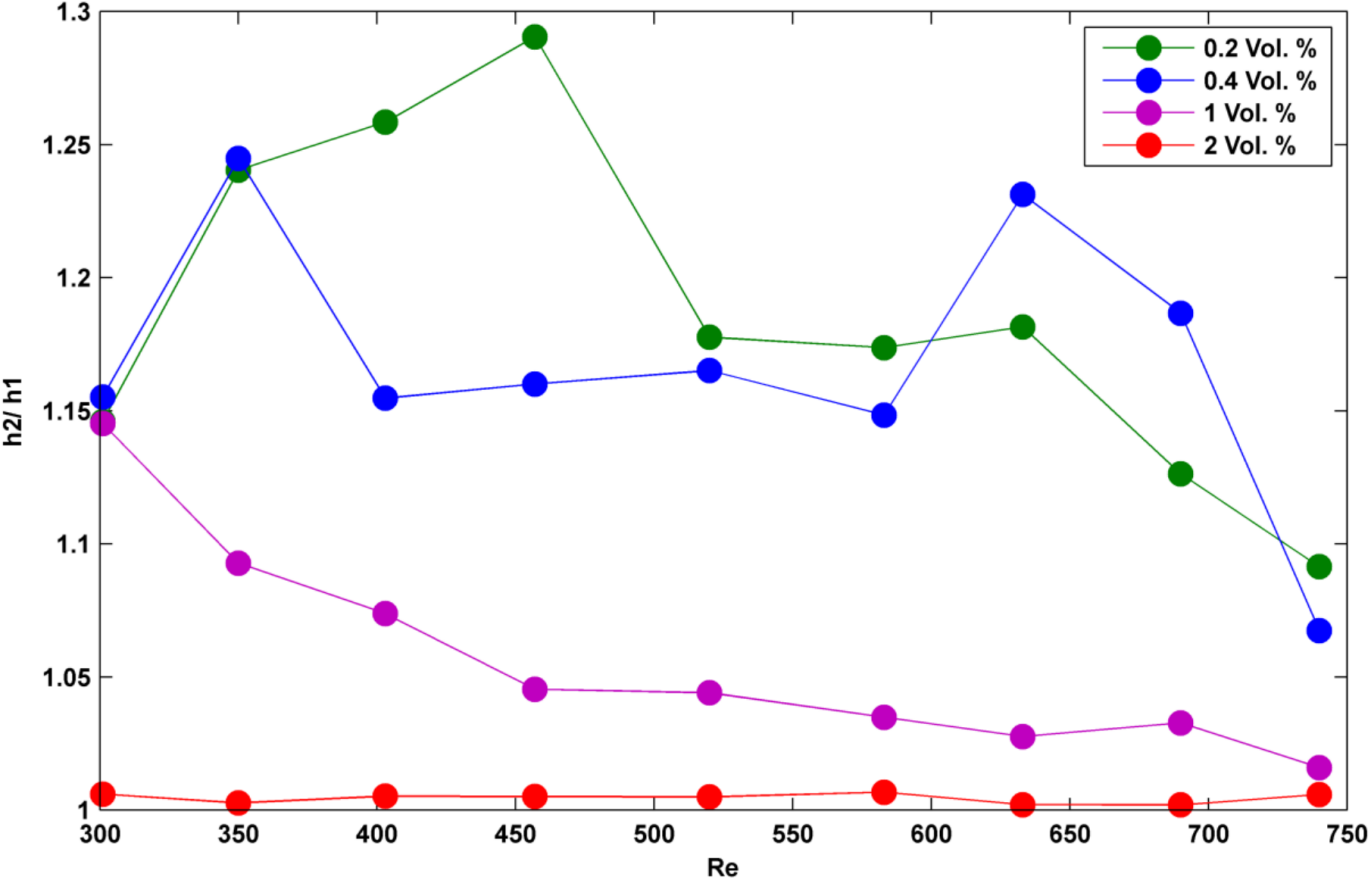
h_2_/ h_1_ in different Reynolds numbers and for higher volume concentrations of nanoparticles.

To start with the difference between the two applied geometries, it is worth to note that the heat transfer surface for the two tubes was in the same size; so as it can be seen from Table 1 and also Figs 8 and 9, convective heat transfer coefficient for all the studied test cases (different Reynolds numbers and volume concentrations) was higher for the semicircular tube. Based on the previous discussion regarding the selection of a certain hydraulic diameter for the two tubes and also noting the equivalence of heat transfer surface for the two manufactured tubes, it may be promising to ensure that the possible reasons behind the reported results could be found through the Brownian motion mechanism. So, seemingly, the Brownian motions are more considerable in the semicircular tube than the rectangular one. As it is shown in Figs 8 and 9, h_2_/ h_1_ (note that h_2_ is for the semicircular tube and h_1_ is for the rectangular tube) is greater than one for all the Reynolds numbers and volume concentrations. But, also based on Figs 8 and 9, h_2_/ h_1_ increases by addition of nanoparticles into the base water up to vol. 1% and also this factor increases up to a certain Reynolds number and then decreases with the increase of Reynolds number. As the matter of fact, deciphering the nature of Brownian motion is still of the controversial challenges among many researches and for the models it is often mathematical convenience, rather than the accuracy of the models, that motivates their use. And it is worth to note that a more general aspect for simulating the Brownian motions is to assume that the huge number of bombardments a Brownian particle undergoes will lead to a homogenous distribution of the particles in which Einstein first assumed that the density of each position in the next interval of the Brownian motion is mainly resulted from the density of all positions (with considering a probability function) in the previous interval and consequently, he reached a diffusion- like equation for Brownian motion. But yet, there is no proof that this assumption is compact and absolutely compatible to the nature. Furthermore, Brownian motions have been also modeled by the available experimental data as for the nanofluids the effective viscosity and thermal conductivity of the suspended nanoparticles into the base fluid can be divided into a static effect and a dynamic one in which the effect of Brownian motions can be mainly found through the dynamic effect [19]. In those models, the effect of Brownian motions is described by the effects of volume concentration and local temperature. Therefore, it may be understood that the topological behavior of Brownian motions is mostly reliant on volume concentration and temperature. It is true that for a given volume concentration topology may breaks down subject to the thermal conditions but the question is that are we truly sure that there is a certain topology when the two factors of volume concentration and temperature are fixed? Regarding this, there are some works on the boundary (geometry) effects on Brownian motions [20]. So, even in a certain volume concentration and thermal conditions, we may have different behavior of Brownian particles (ex. nanoparticles). Basically, a full simulation of the present problem can be relatively done based on Monte- Carlo analysis (mainly for including particles aggregation) which normally deals with the probabilistic (stochastic) estimations for the particle path lines. But yet, there might be other alternative methods for estimating the effect of Brownian motions.

Based on the outcome results of the present work, the Brownian motions might more increase through the semicircular tube than the rectangular one. As mentioned, this increase almost falls down in certain Reynolds numbers for each volume concentration. So, it seems that although the fluctuation terms of the velocity components rise with the increase of Reynolds number and hence the Brownian motions of the nanoparticles (due to the increase of turbulence fluctuations), but these extra probabilistic motions negatively affect the performance of semicircular tube. We hereby make an assumption helpful to simplify studying the behavior of Brownian motions. We assume that the effectiveness of Brownian motions on convective heat transfer coefficient is mostly relied on the number of collisions between the nanoparticles and the heated surface. Besides, it is known that eddy viscosity is basically depended on a contribution of kinematic energy and dissipative energy of the fluctuation terms. Further we know that eddy viscosity is greater at the center of the tubes due to the highest kinematic energy and the lowest dissipation. Subsequently, the increase of Reynolds number affects mostly the central Brownian motions. Reminding the assumption (enhancement of h, is reliant on the number of nanoparticle collisions to the heated surface), the increase of central Brownian motions could have mostly affected the particle interactions at the center of the tube not the collisions to the heated surface! (Please note that this conclusion might be rather radical because it is somewhat in the opposite of the general aspect towards the Brownian motions which states Brownian motions will always result in a homogeneous distribution over the continuous intervals but for this case, maybe the size of nanoparticles have been smaller than the eddies and so nanoparticles could not penetrate the laminar sub- layer due to Brownian effect).

Moreover, performance of the two applied tubes approaches to each other as the volume concentration increases. This could mean that by addition of extra nanoparticles, the maximum collisions (especially between the nanoparticles and the heated surface) might have reached an equal value; so there would be no difference in the performance of the two tubes in enhancing convective heat transfer coefficient. It must be also cited that this equivalence was observed in 2% vol. for the specific applied nanofluid (Ag/ Water); so this value may change while using different nanofluid types. To be more specific, as we have used some certain volume concentrations of Ag/ Water nanofluids in the present experiment, this equivalence might even have occurred in a certain volume concentration between 1% and 2% vol. (see Fig 9).

Moreover, it is still doubtful to relate the effect of Brownian motions on h only to the collisions of nanoparticles to the heated surface. But we hypothesize that it may be possible to divide the manipulation of h due the presence of nanoparticles into two parts in which one is the static part (which can be directly obtained by considering the manipulation of thermo- physical properties relatively through Maxwell and Einstein relations) and the second is the dynamic part which adds the effect of Brownian motions through an analytical approximation directly to the static part of h. this analytical approximation will be based on a comprehensive possibility analysis which the particles (nanoparticles) collide and/ or approach to the heated surface also with including the possible particle interactions (we will also try to consider each collision to the heated surface as a momentary expansion of the heated surface). Seemingly, there is not a direct mention to the working temperature of the particles in our future model. But a more careful consideration of the present problem may encourage one to conclude that the working temperature might have nothing to do with the topological behavior of the many bombardments Brownian particles will undergo (mainly when the temperature is distributed over a similarity scale) and so the working temperature may cause a fast repetition of the probabilistic algorithm. Because, actually probability function for Brownian motions can be applied regardless of the working temperature. But as the convective heat transfer coefficient is related to time (remind the dimension of h), the working temperature will certainly also play role. Based on the provided discussion, in the close future work, we will try to develop a simple analytical approximation model for including the effect of Brownian motions on convective heat transfer enhancement and compare it to the stochastical simulation (Monte- Carlo analysis) and validate it with some experimental data for different nanofluid types. Another interesting thing that was noticed during the experiment is that it might be also hypothesized that the boundary effect (geometry effect) on Brownian motion can be studied through a symmetry-like function. The reason for this hypothesis may be explained by considering this fact that the semicircular tube (with one symmetry axis) has shown a higher performance in convective heat transfer coefficient rather than the rectangular one (with two symmetry axis) under the conditions of the present experiment. Therefore, it may be understood that as the symmetry axis increases, particles (nanoparticles) may behave within a higher order and possibly not intended to undergo more Brownian motions (as if Brownian motions happen for reaching an order! (referred to the aspect of universal order)). Further understanding through the symmetry perspective is still under progress.

## Conclusion

In the present study, two different schemes for the cooling system of photovoltaic cells were compared. Coming to this point that based on the presented discussion in this work, milli and micro heat sinks with parallel rectangular-formed graves have proved themselves efficient in removing heat from PV cells, it was aimed to introduce a new promising scheme for manufacturing these graves. So, a semicircular tube was studied in order to be compared with the rectangular one in the term of convective heat transfer coefficient. This comparison was done in different Reynolds numbers (301 to 740) and for seven different volume concentrations of Ag/ Water nanofluids (0.03%, 0.07%, 0.1%, 0.2%, 0.4%, 1% and 2%). According to the outcome results of the present experiment, convective heat transfer coefficient increased by addition of the nanoparticles for the two geometries and for all the applied volume concentrations. But this increase differed from the two tubes as shown within the present study, semicircular tube had the advantage over the rectangular one in all the studied cases. As discussed, because of using the same hydraulic diameter and heated surface for the two tubes, it was promising that one could seek for the possible reasons behind the results through the aspect of Brownian motions. By taking the boundary effect on Brownian motions into account, a discussion was provided to justify the outcome results of the present work. We hereby hypothesize that boundary effects on Brownian motions may be considered as an independent invariant which affects the Brownian patterns regardless of the nanoparticle concentrations and temperature distribution. As discussed, examining this hypothesis is still in progress. In further works, we will try to develop an analytical approximation to include the effect of Brownian motions as an external dynamic pattern in convective heat transfer coefficient by the means discussed through this paper.

## Supporting information

S1 Appendix This file presents appendix. (DOCX)
