# Supplementary material for: A Comparison between heat transfer performance of rectangular and semicircular tubes considering boundary effects on Brownian motions in the presence of Ag / water nanofluids: Applicable in the design of cooling system of photovoltaic cells
Source: PLoS One. 2017 Jul 28;12(7):e0180883. doi: 10.1371/journal.pone.0180883 (PMC5533334; doi:10.1371/journal.pone.0180883)
Supplement: S1 Appendix — (DOCX) [file pone.0180883.s001.docx]

**Appendix:**

The independent T- Test for friction factor of semi-circular and rectangular tubes: (Derived from SPSS software)

| **Group Statistics** | | | | | | | | | | | | | | | | |
| --- | --- | --- | --- | --- | --- | --- | --- | --- | --- | --- | --- | --- | --- | --- | --- | --- |
|  | | VAR00001 | N | | Mean | | | Std. Deviation | | | Std. Error Mean | | |  |  |  |
| VAR00002 | | 1 | 7 | | 1.0154 | | | 0.01127 | | | 0.00426 | | |  |  |  |
|  |  | 2 | 7 | | 1.0166 | | | 0.01156 | | | 0.00437 | | |  |  |  |
| **Independent Samples Test** | | | | | | | | | | | | | | | | |
|  |  | | | Levene's Test for Equality of Variances | | | t-test for Equality of Means | | | | | | | | | |
|  |  | | | F | | Sig. | t | | df | Sig. (2-tailed) | | Mean Difference | Std. Error Difference | | 95% Confidence Interval of the Difference | |
|  |  | | |  |  |  |  |  |  |  |  |  |  |  | Lower | Upper |
| VAR00002 | Equal variances assumed | | | .005 | | 0.945 | -0.208 | | 12 | 0.838 | | -0.00127 | 0.00610 | | -0.01457 | 0.01202 |
|  | Equal variances not assumed | | |  | |  | -0.208 | | 11.993 | 0.838 | | -0.00127 | 0.00610 | | -0.01457 | 0.01202 |
